# Effectiveness and learning experience from undergraduate nursing students in surgical nursing skills course: a quasi- experimental study about blended learning

**DOI:** 10.1186/s12912-023-01537-w

**Published:** 2023-10-20

**Authors:** Yan Ran Li, Zong Hao Zhang, Wen Li, Pan Wang, Shu Wen Li, Dan Su, Ting Zhang

**Affiliations:** School of Nursing, Anhui Medical University, No.15 Fei Cui Road, Hefei, 230601 Anhui China

**Keywords:** Blended learning, Nursing undergraduate, Skill performance, Learning engagement, Learning experience, Mixed study, Quasi- experimental study

## Abstract

**Background:**

Blended learning is increasingly being adopted, and yet a gap remains in the related literature pertaining to its skill performance, learning engagement and inner experience in undergraduate surgical nursing skills course.

**Objectives:**

To investigate the changes in skills performance and learning engagement in the application of blended learning, and what it actually brings to nursing students.

**Design:**

The study uses a historical control, two-armed, mixed and quasi-experimental design.

**Methods:**

The blended learning version of the course was offered to the 2019 class of 334 nursing undergraduates. Quantitative and qualitative data were collected after the course to obtain a comprehensive understanding of the course effects compared with the 304 nursing undergraduates of grade 2017 who adapted traditional learning. Quantitative data were analyzed by descriptive and inferential statistics using IBM SPSS 26.0, and qualitative data were encoded using Nvivo11.0.

**Results:**

There were significant differences in skill performance and learning engagement between the class of 2017 and 2019 (*p* < 0.001). Combined with further analysis of the interview data, 3 first-level nodes and 8 secondary nodes were determined. Students’ opinions, comments and suggestions on the application of blended learning are refreshing.

**Conclusion:**

Moving forward with blended learning: opportunities and challenges go hand in hand. Researchers need to continually modify their research designs to respond to variable educational environments.

**Supplementary Information:**

The online version contains supplementary material available at 10.1186/s12912-023-01537-w.

**Table:** 

Text box 1. Contributions to the literature	
• There is an increasing debate on how both traditional and online education can be combined for effective teaching. Fewer studies on blended learning from the perspective of learning engagement and students’ internal experience. This study fills a gap in previous research • The study uses a qualitative and quantitative synergetic study design. Realizing a truly student-centered philosophy	

## Introduction

The traditional teacher-centered teaching patterns have been the mainstay of nursing education [1]. Traditional learning refers to the traditional method of education in which students enter a specific place at a specific time for face-to-face interaction with teachers and classmates [2]. The schedule and content of the course will be assigned by the teachers, and the classroom serves as the primary learning environment. It typically involves lectures, textbook readings, note-taking, and paper assignments [3]. Circulating knowledge is a one-way street in traditional teaching: teachers act as transmitters of knowledge and students are passive recipients [4]. The limitation of time and space greatly hinders students' study. Teaching drawbacks such as a single form of teaching, limited teaching activities, and poor student initiative and motivation have not been solved over time [5]. The emergence of online learning has, to a certain extent, facilitated the application of quality teaching resources and the diversification of education [6]. However, It has to be admitted that complete online learning isn't flawless either. For instance, users must have a high degree of self-control in order to take advantage of the benefits of online learning [7]. It's not always the way it should be. A study showed that coursework completion rates for single online instruction were less than 10% [8]. It has been recognized that neither singular online teaching nor traditional teaching can achieve specific pedagogical goals [9]. Even though traditional teaching has many drawbacks, there is no substitute for face-to-face teacher-student interaction and high-frequency collision of ideas in the classroom [10]. There is a mounting debate on how to combine traditional and online education for effective teaching and learning [11]. Blended learning formats will be the inevitable orientation of education in the future [12].

Blended learning takes multiple formats and can be elusive to define. There are primarily four interpretations relevant to blended learning [13]: (1) blending based on web-based technologies; (2) blending of various teaching methods; (3) combination of any form of instructional technology with face-to-face instruction by the instructor; and (4) integration of instructional technology with work tasks to form an effective combination of learning and work. The definition employed in our study is strongly interrelated with the third point above. Blended learning is described as a combination of traditional learning and a range of pedagogical approaches supported by information and communication technology [14]. This definition encompasses blended learning, distributed learning, decentralized learning, hybrid learning, and flexible learning [15]. Rather than a simple convergence of technologies in the true sense of the word, it creates a student-centered pedagogical model that combines the advantages of online learning (e.g., flexibility, wide range of educational resources, timely updating, and resource sharing) with the interactivity of traditional teaching [16]. In this pedagogical approach, students engage in both offline classroom activities and online learning experiences. They may participate in lectures, discussions, and collaborative work in the physical classroom, while also utilizing digital resources, video lectures, interactive modules, or online quizzes for learning [17]. Online learning content is usually delivered through a learning management system or other digital platforms. Blended learning allows students to access course materials at their convenience, review content as needed and engage in interactive online learning activities [18]. At the same time, this approach allows teachers to personalize instruction, track student progress and provide additional support [19].

The novel coronavirus disease (COVID-19) pandemic has challenged nursing educators worldwide [20]. One of the goals of nursing education has been to accelerate the training of future nursing workers to respond to future public health emergencies. However, in local medical universities in developing countries, including China, school facilities and faculty numbers are not sufficient to match the rapid growth in enrolment [21]. Surgical nursing is one of the main courses in undergraduate nursing education. Besides a solid theoretical knowledge, it requires students to master a wide range of surgical nursing operations. However, traditional teaching methods are constrained by time and space, and not all students have the opportunity to participate directly in practical demonstrations, and relying solely on abstract verbal explanations and photographic displays, students often fail to grasp all the operation points. Therefore, we urgently seek an innovation in education to salvage the current situation.

Blended learning has already practiced in medical disciplines and its effectiveness has been verified [22]. "Effectiveness" generally refers to student performance in final examinations [7]. Blended learning strategies strengthen nursing students’ academic performance compared to traditional teaching in studies involving undergraduate nursing students [23]. However, it is debatable whether blended learning can improve the operational performance among undergraduate nursing students. A study found that blended learning is more effective than traditional teaching in terms of skill performance [12], but a meta-analysis showed some improvements in skills compared with traditional learning, but the difference was not statistically significant [24]. Hence it is not yet certain whether the integration of Information Technology (IT) into the traditional classroom will actually boost students' performance in surgical skills.

Getting to know how engaged students are with their learning is vital to assessing blended learning. Learning engagement is defined as a positive, fulfilling, and work-related state of mind characterized by vigor, dedication, and absorption [25]. When students are more engaged in their learning, they are more motivated, focused, and committed to achieving their goals [26]. This results in enhanced academic performance, higher retention rates, and greater academic satisfaction, conversely, when students are less engaged or less fully invested in their learning, they may struggle to learn new things, lose interest, or even drop out of classes [27]. The pedagogically orientated use of educational technology can superiority support student engagement compared to traditional learning [28]. Researches suggested that it may be related to increased independent learning, autonomy and interest in learning [29–31] Under these positive influences, students are motivated to follow the course rules, devote themselves to the course, take action to solve the learning problems and achieve learning success, which in turn leads to new emotional experiences, creating a virtuous cycle that enhances learning outcomes [32]. The indicator of learning engagement laterally reflects the effectiveness of blended learning. Compared to cold numbers, It's more "humane.", which pays more attention to the state of student learning and compensates for the deficiencies of numbers assessment [33]. Currently. There are few studies that directly point out that blended learning can increase student engagement.

What exactly does blended learning bring to students is the highlights of our research. The fundamental purpose of blended learning is to serve the students and We are more interested in discovering how students experienced the education model, such as cognition, attitude and opinions. Satisfaction is the most common indicator used by students to evaluate course teaching. Blended learning has been found to increase student satisfaction [34], however the use of satisfaction alone as an indicator is not comprehensive enough and there is no authoritative quantitative tool available to students for course evaluation. The introduction of qualitative research methods can do us well in addressing these issues. The mixed methods to research, combining quantitative and qualitative aspects, can maximize our understanding of the effectiveness and experience of blended learning among students [35]. At the same time, qualitative methods can compensate for quantitative gaps [36].

The main purposes of this study were to compare the skill performance and learning engagement of nursing students with different learning modes by adopting a quantitative methodology, and exploring the innermost experiences of blended learning users through qualitative research. These findings will help to provide lessons and implications for nursing education programs in universities worldwide, while maintaining and safeguarding the well-being of students.

## Methods

### Study design

**Fig. 1:**
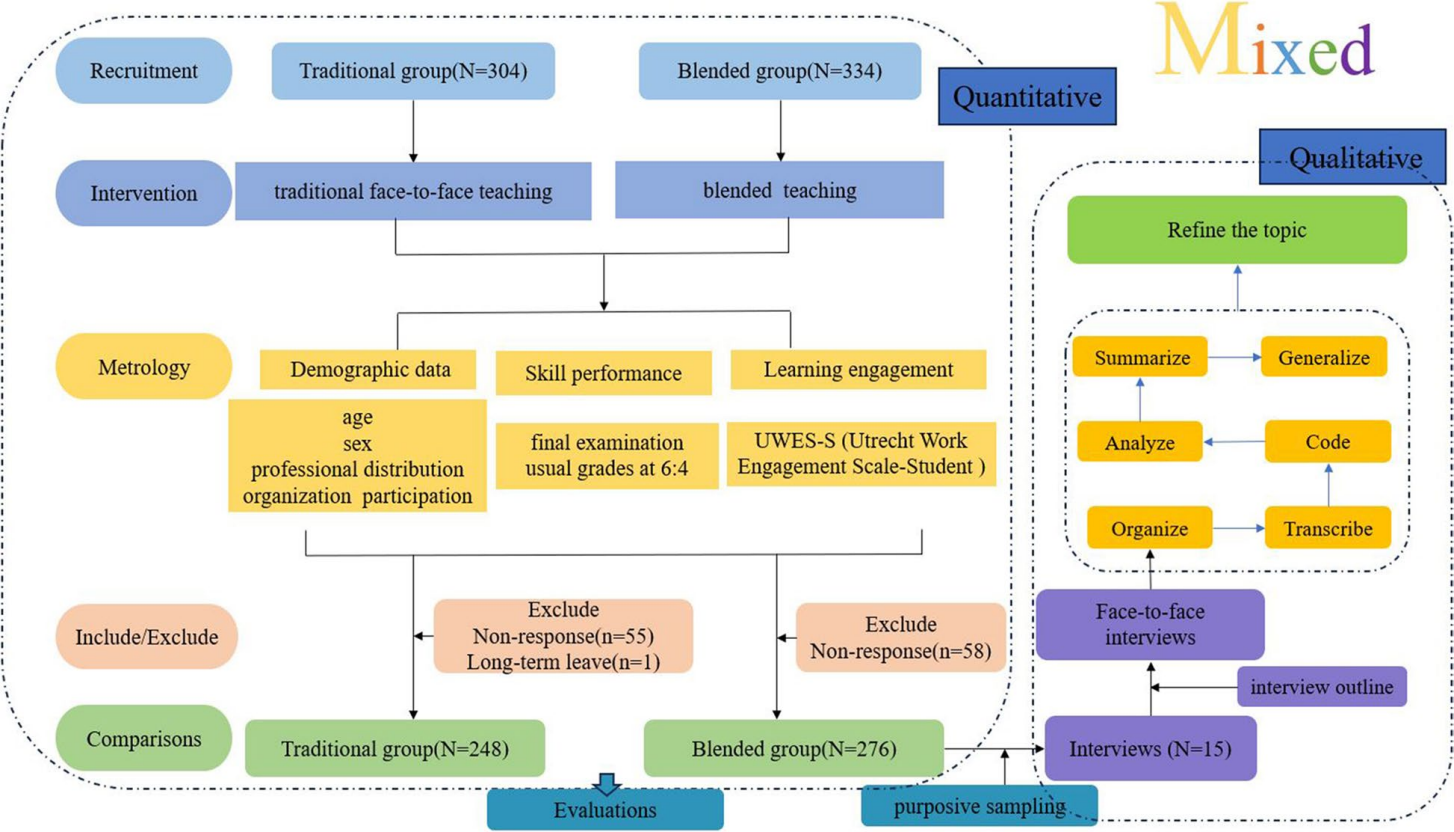
Flowchart of experimental design

The study uses a historical control, two-armed, mixed and quasi-experimental design. The experiment was conducted in different semesters; we implemented traditional teaching in 2019 and blended learning in 2021, and the study was conducted on third-year undergraduate nursing students with different entry dates. Quantitative data were collected on skill performance and learning engagement assessment scales, and qualitative data were collected by interviewing blended learning users. The detailed study design is illustrated in Fig. 1.

### Sample size and estimated study power

G power 3.1 was used to calculate the sample size. To maximize statistical power, the sample size estimate was calculated with a mean effect size of 0.5 for the outcomes, an alpha set at 0.05, and a power of 0.80, and the result showed a requirement of 64 nursing students for each group [37]. A minimum of 80 participants per group were eventually enrolled, taking into account a 20% dropout rate.

### Participants and recruitment

This study was conducted among undergraduate nursing students in different grades at a public medical university in China. First, a method for cluster sampling was adopted. A total of 304 nursing students from the class of 2017 were invited to attend a traditional surgical nursing skills course. A total of 334 nursing students from class of 2019 were invited to participate in the blended learning. Second, after the end of the course, according to gender and skill performance, 15 nursing undergraduates in Grade 2019 were selected for individual face-to-face interviews by purposive sampling. The specific inclusion criteria were as follows: 1) Full-time nursing students enrolled in the national unified examination; 2) Participants providing informed consent and voluntarily participating in this study; 3) Participation in the course to apply for leave and suspension from school, with a cumulative leave time of not more than 1 month; and 4) Normal cognitive and behavioral abilities.

### Blended teaching program

Eight surgical nursing skill classes were offered to the 2017 undergraduate nursing students in 2019, all of which used traditional face-to-face teaching methods. The blended learning version of the course was offered to the 2019 class of nursing undergraduates in 2021, which combined synchronous and asynchronous online learning modules on the basis of traditional face-to-face teaching.

On the basis of the original course, 10 new chapters of online learning are added, and the original 8 traditionally delivered chapters are integrated into the online content to form a unique blended version of the teaching arrangement. The online learning module was developed using a variety of authoring software tools and screen play (pre-recorded), which were accessible through the learning management system. The same team offered both courses. The specific blended course arrangement is shown in Additional file 1.

**Fig. 2:**
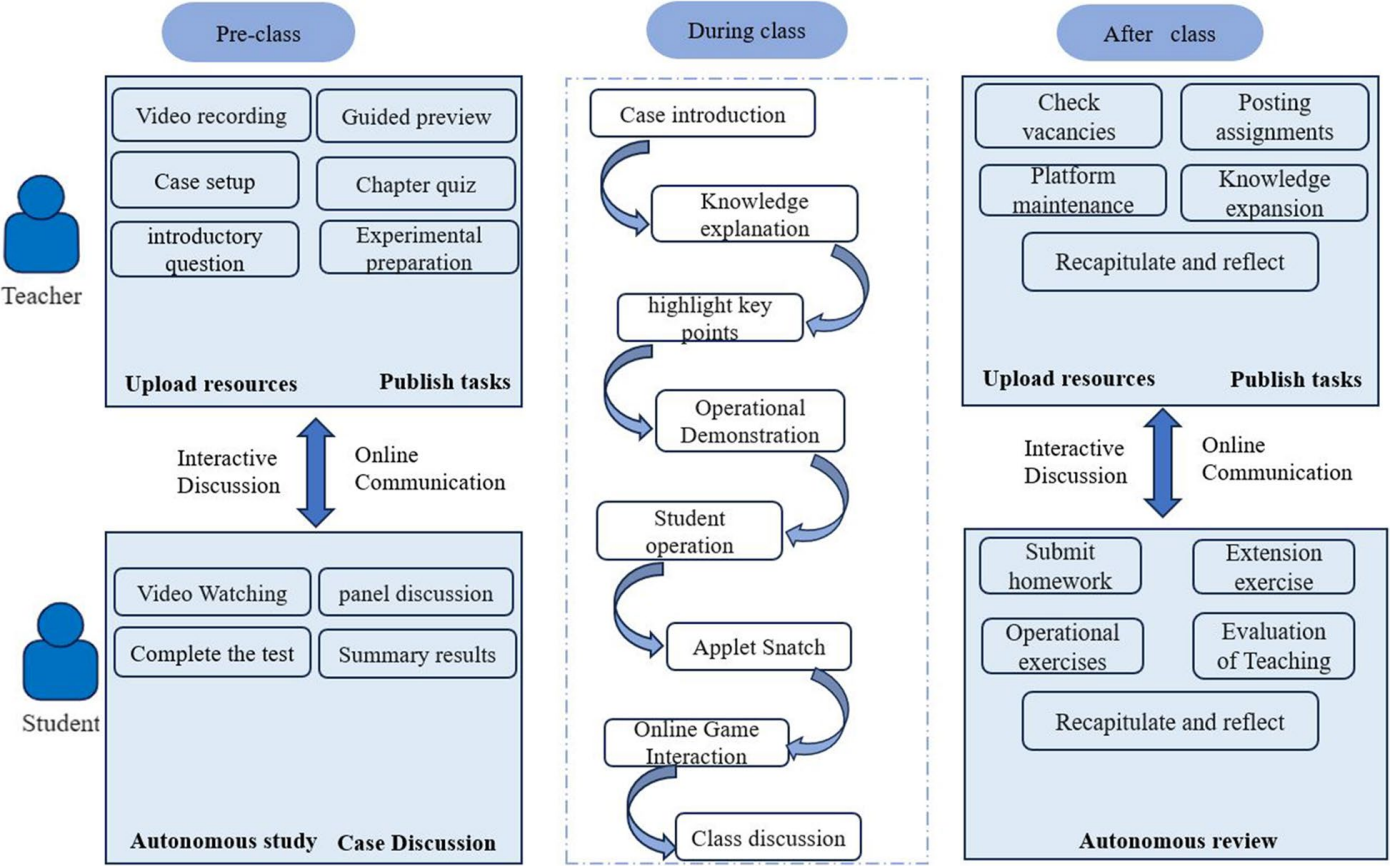
Blended learning operation chart

According to the characteristics of the content of the Surgical Nursing Skills course, the blended classroom teaching method of Preview before class—Classroom teaching—Review after class was established to help students understand and accept the content of the course and achieve satisfactory learning outcomes. An example is the care of a closed chest drain:

- Preview before class: The instructor publishes a pre-recorded video on closed chest drainage through the platform, with 2–3 quiz questions interspersed in the short video. Students need to log in to the learning platform to complete the video and chapter quizzes before the offline course. In addition, teachers can set up clinical scenarios through the platform to introduce cases and inspire students to think. Students are free to form groups and complete single or team discussions.
- Classroom teaching: the teacher explains the purpose, significance and etc. of closed chest drainage to students through clinical cases, and analyses the key and difficult parts in detail. In the teaching process, the learning platform is used for teacher-student interaction, such as setting up a quiz, random drawing and other special activities to enrich the teaching content. After the theoretical study, the teacher personally demonstrates the skill operation in the classroom and invites students to make a retest.
- Review after class: Teachers release post-class assignments, such as online discussion on "What to do if the closed chest drain falls off", and track students' learning through the platform. Uploading extended resources to bridge the gap between clinical and actual teaching, as well as maintaining the operation of the platform. Students submit reports, complete discussions, practice operations on their own, and complete teaching evaluations. See particular in Fig. 2.

### Instruments

The quantitative survey collected demographic data, skill performance, and learning engagement. Demographic data included age, sex, professional distribution and organization participation.

Skill performance was determined by the proportion of final examination results and usual grades at 6:4, which were directly derived from the official platform of the university.

Learning engagement was measured using UWES-S (Utrecht Work Engagement Scale-Student scale) compiled by Schaufeli (2002) [25]. The scale consists of four dimensions and seventeen items, of which vigor and absorption include six items each and dedication includes five items. The scale was scored on a 7 points Likert-scale. The Cronbach’s α was 0.87.

The qualitative survey mainly adopted a self-made interview outline, and conducted one-on-one face-to-face interviews with 15 nursing undergraduates who used blended learning, around the six questions in the outline.

### Trustworthiness

First of all, the researchers themselves are an invaluable tool in qualitative research. The researchers have received professional training in qualitative research courses during their school years, as well as learning related to qualitative research data collection, and have conducted pre-interviews under the guidance of their supervisors, mastering semi-structured in-depth interviewing techniques, data collection methods, and analytical methods. Second, relevant literatures according to the purpose of the study were explored, initially formulated the interview outline, and revised the outline after consulting four members of the Surgical Nursing teaching and research team (including one professor, one associate professor, and two lecturers); two students who met the criteria were selected to be pre-interviewed, and the interview outline was finally formed after the discussion of the research team. Third, the researchers, as students at the same school, joined the QQ group used for classroom management while the blended learning was underway. Not only did this allow the researchers to observe students’ responses in a timely manner, but it also facilitated the establishment of long-term relationships and shortened the length of the freeze-breaker required at the start of the later interviews. Finally, callbacks to interviewees is also a step towards ensuring the quality of qualitative research. After the interviews were completed and the recordings were converted into text, we confirmed the accuracy of the interviews with the interviewees through an online chat and asked them to evaluate the interview process for later improvement.

The specific interview outline is as follows:

1. Compared with the traditional learning mode, after a semester of blended learning, which one do you prefer? Give a reason.
2. Does the model of blended learning meet your needs for learning skills?
3. What do you think blended learning approach has brought to you?
4. What was your greatest feeling during the learning process?
5. Please rate the online and offline blended teaching mode from 0–100. Do you hope your younger brothers and sisters continue this learning method in the next semester?
6. Do you have any other suggestions or opinions?

### Data collection

Through the ‘questionnaire Star’ platform, the demographic questionnaire and the UWES-S were compiled into an electronic version of the questionnaire. After obtaining informed consent from the participants, the participants filled out the questionnaire by ‘scanning the code’ which was distributed to the QQ group. The first survey object of the electronic questionnaire was undergraduate nursing students of Grade 2017 in 2020: a total of 304 questionnaires were sent out, of which 248 were effectively received. The second survey object was undergraduate nursing students of Grade 2019 in 2022: a total of 334 questionnaires were sent, and 276 valid questionnaires were collected. The scores of surgical nursing skills of two grades were obtained through the educational administration system and the rain classroom platform.

A phenomenological qualitative research method was adopted. Based on the interview outline, the researcher conducted a one-to-on in-depth interviews with 15 undergraduate nursing students of Grade 2019 face-to-face at the end of the course. Each time, the length was approximately 60–90 min, ambient noise was eliminated, and privacy was maintained. When dealing with the interview materials, the names were handled anonymously and replaced by a serial number of the English alphabets A–O.

### Statistical analysis

Data were analyzed using the IBM Statistical Package for the Social Sciences (Statistics) version 26.0. Descriptive statistics of mean, standard deviation, and percentage were used for demographics, skill performance, and other study variables. independent t-test was used to examine the differences between blended and traditional learning methods in relation to students’ skill performance and learning engagement. Statistical significance was set at *P* < 0.05. The interview data were imported into Nvivo11.0 in the form of word text, and encoded in open, associated, and selective modes. When the 12th interviewee was being interviewed, we found that no new coding content appeared with the input of word text materials, and three more interviewees were interviewed to verify whether the data had been saturated. We also rechecked the codes of interview materials, the node closest to the research topic was selected.

## Results

### Demographics

Two hundred and forty-eight nursing students from Grade 2017 were enrolled as the traditional learning group, including 65 males and 183 females, with an average age of 20.42 ± 0.84 years, 276 students from the Grade 2019 were the blended learning group, including 53 males and 223 females, with an average age of 20.57 ± 1.11 years. A total of 154 nursing students in the traditional study group were transferred to the nursing program by their majors, and 56 students were class officers and were able to actively participate in various activities. In the blended learning group, 183 students were transferred and 68 were class officers. The baseline data of the two groups were consistent and comparable (see Table 1 for details). We ensured a rigorous experimental design as much as possible, in addition to comparable demographic information. In the teaching and learning process, the faculty, the school district environment, and the use of teaching materials were identical. At the same time, we used the evaluation indicators: grades, test paper questions drawn from the same question bank.

**Table 1:** Description and comparison of demographic characteristics, skill performance and learning engagement scores between two groups of nursing undergraduates

**Variable**	Traditional (*n* = 248)	Blended (*n* = 276)	t
	M ± SD / Frequency	M+SD / Frequency	
Age	20.42±0.84	20.57±1.11	*-1.696*
Gender	Female:183	Female:223	
	Male: 65	Male: 53	
Professional Distribution	Yes:154	Yes:183	
	No: 94	No: 93	
Organization Participation	Yes:56	Yes:68	
	No: 192	No: 208	
Skill Performance	76.29±8.06	81.19±5.86	-8.723**
Vigor	16.96±6.85	28.99±5.43	-22.094**
Dedication	14.91±6.06	22.84±4.83	-16.428**
Absorption	16.85±6.97	27.84±5.49	-19.889**
Learning Engagement	48.73±18.18	79.67±14.99	-21.106**

*M* mean, *SD* standard deviation

^*^ *p* < 0.05, Statistically difference

^**^ *p* < 0.001.Statistically significant difference

### Skill performance and learning engagement

**Fig. 3:**
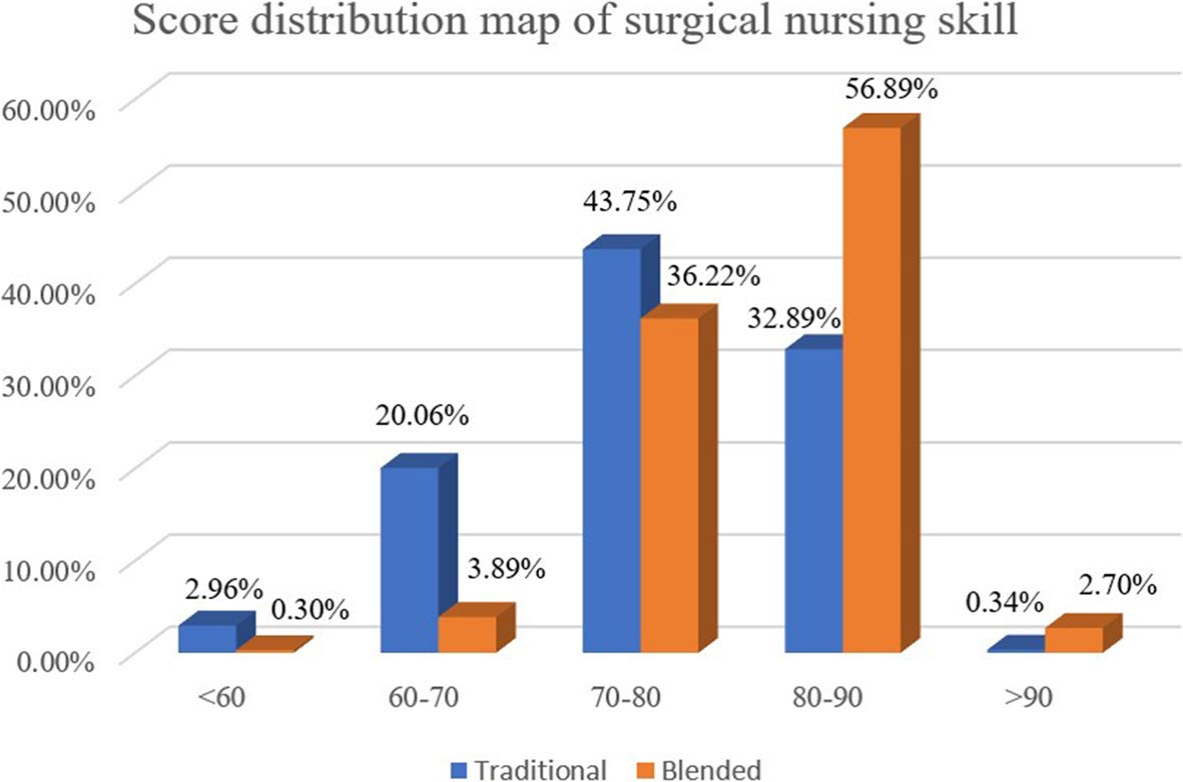
Score distribution map of surgical nursing skills

The final course grades were obtained from the university’s educational administration management system and the classroom platform. Table 1 shows the overall score of the blended group (81.19 ± 5.86) was better than traditional group (76.29 ± 8.06), with a statistically significant difference (t = -8.723, *p* < 0.001). By comparing the results of the bar charts (Fig. 3), It can be seen that the integration of IT into traditional education has resulted in a decrease in the proportion of students in the lower band (skill performance < 70) and an increase in the proportion of students in the higher band (skill performance > 80). There is a tendency for students in almost every mark range to migrate to higher mark levels. This demonstrates that blended learning does not only help to improve overall scores by raising the performance of poor students, but also allows students in the higher bands to progress.

According to the data analysis results, the level of learning engagement of nursing students in blended (79.67 ± 14.99) was higher than traditional (48.73 ± 18.18), there were statistical differences in the overall score of learning engagement and scores of all dimensions between the two groups (t = -21.106, *p* < 0.001) (see Table 1 for details).

#### Data analysis of students’ cognition, attitude, and opinions

A purposive sampling was conducted among the 2019 Grade nursing undergraduates based on gender and skill performance with a total of 15 students. Among them, the male-to-female ratio was 3:2. The skill scores are representative in all score segments (see Additional file 2 for details).

Combined with the analysis of the interview data, three first-level nodes were determined: 1) Conforming to the development of the times, reflection and worry about the blended learning, 2) The positive and negative changes that blended learning brings to students, and 3) Students' comments and suggestions on blended learning. In addition, there were 8 secondary nodes: 1) cause of occurrence, 2) reflection and worry, 3) advantage and disadvantage, 4) benefits and obstacles,5) satisfaction,6) platform usage, 7) resource updates, and 8) faculty capacity enhancement. The original information in the literature is the three-level node, namely, the reference point, which is the encoded text content in the qualitative data.

### Theme 1: conforming to the development of the times, reflection and worry about the blended learning

Students affirmed that the emergence and development of blended learning is a major trend. Moreover, they thought about and looked forward to the prospects of blended learning.

A: This is in line with our development, that is, the progress of consistent development of the nursing discipline, which is also why blended learning can emerge. …

K: Offline platforms have been unable to meet the learning requirements of contemporary college students, which is the reason for the emergence of online platforms. …I really look forward to it. Will the future be a fully digital era !

O: What about our model after the end of the pandemic? What is suitable for the development of a society? Will it always change according to the needs of students? …

### Theme 2: the positive and negative changes that blended learning brings to students

The students had a clear understanding of the advantages and disadvantages of blended learning. blended learning can meet the learning needs of students and make students have a sense of benefit and pleasure. While it also hinders their progress to a certain extent.

I: We can review knowledge repeatedly and learn the knowledge points more clearly and systematically. Videos and online materials are more convenient to review. Meanwhile, the online information is complete, mainly very authoritative, and sent by the teacher.

G: We have a little worry about online learning could to become a burden on students. E-learning requires high power consumption of electronic equipment and network fluency. when you watch the video, you will also be disturbed by some information from the outside world. A higher demand for autonomy may lead to a lower learning efficiency.

H: In traditional learning, the teacher sometimes speaks too fast to record or understand. the blended learning model can meet my learning needs in surgical nursing. I think that my study has made progress. My operations are more standardised, and my knowledge is more solid. …

### Theme 3: students’ comments and suggestions on blended learning

The students clearly expressed their love for blended learning with a high score of satisfaction and expressed hope that the next class of students will continue this mode. they put forward their own suggestions on blended learning from platform usage, resource updates, faculty capacity enhancement.

A: Here, I will take Sudan, one of our excellent teachers, as an example. I hope that other teachers can learn from her in the future, develop unique teaching styles, and improve their teaching ability.

B: The video materials still need to be constantly updated, and I still feel that there is still a gap between the requirements and some specific clinical operations. …

F: I also feel that blended learning is very good, with a satisfaction rating of 98. I hope that the younger brothers and sisters of the next semester will continue this learning method.

O: As I mentioned earlier, how can we diversify resources? Not just by videos, such as bibliographic indices, but the entire resource platform. It lacks a unified guide for use. … Our classmates may not use the electronic resources of e-learning platforms much as they are more accustomed to the original teaching methods sometimes.

## Discussion

This study found that nursing students’ skill performance significantly improved after adopting blended learning. Most available studies suggest that blended learning is more effective in knowledge acquisition than traditional learning [38]. The nurse students strengthen their clinical skills by repeatedly watching clinical videos to establish a connection between theory and practice anytime and anywhere according to their own rhythm [29]. Blended learning fully considers the individual differences of students, and does not evaluate students’ academic performance purely by examination scores [39]. It adds several evaluation indices, such as online task completion, homework completion, and classroom participation, which can objectively and multi-dimensionally evaluate students’ learning performance. Completion of these indicators will be calculated as the student's usual grade into the final grade (4:6). The results emphasize the portability and overall benefit to student learning of clinical skills by introducing video podcasts into nursing curricula [40].

Undoubtedly blended learning can improve the degree of learning engagement among undergraduate nursing students. Because of the innovation in teaching methods, blended learning overcomes the limitations of classroom teaching, time, and space [41]. Teachers use the online platform for high-frequency interaction and cooperation with students to stimulate nursing students’ interest in this course. In the nursing learning of most medical colleges in China, the traditional learning materials are mostly paper materials and PowerPoint (ppt), but ppt is only displayed in class, making it inconvenient to take pictures or download. Rich and official learning resources and learning activities (such as videos, electronic question banks, group simulation homework, and expanding resources) make the boring traditional classroom more vivid and flexible, which can attract students’ attention and improve students’ interest in learning [42]. Therefore, we have every reason to believe that blended learning can improve students' interest in learning through the reform of teaching methods, thus increasing the frequency and depth of students' entry into learning state, and students who are more engaged in learning are more likely to get better grades.

Qualitative research results bring new findings to blended learning. This study found that nursing students had an excellent sense of learning experience after adopting blended learning. They have a clear understanding of the occurrence and development of a new model and its advantages and disadvantages. Interestingly, students do not think that e-learning replaces traditional teacher-led training, but complements it. Blended learning is more consistent with the learning habits and motivation of contemporary college students [43]. Teachers upload detailed teaching videos through online modules and reinforce students’ memory points through offline classroom consolidation, which is undoubtedly a more official and convenient way to learn. Nursing students can choose to study anywhere any time and create a suitable learning environment according to their own learning habits. Almost all interviewees expressed their love for the application of blended learning in ‘surgical nursing skills’. These findings are consistent with the previous studies conducted by Li and Schaffner [24, 44]. The information environment created by blended learning can benefit nursing students more [15]. The model in the application of surgical nursing skills can help the nursing students learn this course, but there are still some problems, such as lack of detailed operational guidelines and guide learning modules, hope for the latest clinical technology, etc. It is particularly noteworthy that some students have proposed that teachers’ personal abilities and styles are also factors affecting blended learning, which suggests that there is another requirement in the selection of talents for nursing discipline construction in the future. For students to put forward their opinions, nursing educators and technical support staff must adjust in a timely manner. Everything must be student-centered to obtain a better sense of learning experience.

## Limitation

There are some limitations to this study that should be acknowledged. First, the data were self-reported, which also limits the universality of the study results. Second, the participants were third-year nursing undergraduates of different periods in the same university, course changes were made from semester to semester based on advances in technology, student feedback, course evaluations, and the maintenance of academic integrity. Although we did our best to control for variables (e.g., consistent baseline demographics, same faculty, etc.), the utter randomization and pure experimental design were not possible in the surgical nursing skills course. Finally, the long-term effects of this experiment were not continuously tracked. In the future, we should test the results by conducting blended learning in more fields and different populations.

## Conclusion

This study found that blended learning improves student achievement and engagement in learning and attempts to understand students' internal experiences from their perspective, some interesting conclusions were also drawn. It can be seen that blended learning is in fact a teaching strategy with a great deal of potential. However, there are many challenges as well, the chosen model (e.g. flipped classroom or scenario-based teaching), the use of technology, the process of switching between online and offline activities, and the control over the completion of online activities all add to the complexity of this approach. Consequently, moving forward with blended learning: opportunities and challenges go hand in hand.

### Supplementary Information

**Additional file 1. **Content arrangement of the surgical nursing skill courses.

**Additional file 2. **Demographic characteristics for 15 interviewees.

## Data Availability

The data that support the findings of this study are available from the corresponding author upon reasonable request.
