# Supplementary material for: Effectiveness and learning experience from undergraduate nursing students in surgical nursing skills course: a quasi- experimental study about blended learning
Source: BMC Nurs. 2023 Oct 20;22:396. doi: 10.1186/s12912-023-01537-w (PMC10588121; doi:10.1186/s12912-023-01537-w)
Supplement: Supplementary file 1 — Additional file 1. Content arrangement of the surgical nursing skill courses. [file 12912_2023_1537_MOESM1_ESM.docx]

Additional file 1. Content arrangement of the surgical nursing skill courses

| Chapter Name | VERSION | |
| --- | --- | --- |
|  | **Traditional Blended** | |
| 1. Skin preparation before surgery  2. Operating room environment  3. Prepare hands and personnel for sterility  4.Common surgical instruments and surgical cooperation  5. Nursing work in operating room  6. Dressing changing method  7. Skin and wound care  8. Gastrointestinal decompression nursing  9. T-tube drainage nursing  10. Closed thoracic drainage  11. Nursing care of bladder irrigation  12. Traction and nursing  13. Plaster bandage fixation  14. Nursing care of ventricular drainage  15. Nursing care of surgical drainage  16. Nursing care of colostomy after urinary diversion  17. New Progress in surgical operation | | Online  Online  Traditional Blended  Traditional Blended  Online  Traditional Blended  Online  Traditional Blended  Online  Traditional Blended  Online  Traditional Blended  Traditional Blended  Traditional Blended  Online  Online  Online |
