# Supplementary material for: Effectiveness and learning experience from undergraduate nursing students in surgical nursing skills course: a quasi- experimental study about blended learning
Source: BMC Nurs. 2023 Oct 20;22:396. doi: 10.1186/s12912-023-01537-w (PMC10588121; doi:10.1186/s12912-023-01537-w)
Supplement: Supplementary file 2 — Additional file 2. Demographic characteristics for 15 interviewees. [file 12912_2023_1537_MOESM2_ESM.docx]

Additional file 2. Demographic characteristics for 15 interviewees.

| English alphabet code | Gender | | Skill performance | |
| --- | --- | --- | --- | --- |
| A  B  C  D  E  F  G  H  I  J  K  L  M  N  O | | Male  Female  Female  Female  Female  Female  Male  Male  Male  Male  Female  Female  Female  Female  Male | | 80  83  92  84  90  83  78  69  83  69  79  77  88  89  85 |
